# PARP2 promotes inflammation in psoriasis by modulating estradiol biosynthesis in keratinocytes

**DOI:** 10.1007/s00109-023-02338-z

**Published:** 2023-06-23

**Authors:** Dóra Antal, Ágnes Pór, Ilona Kovács, Katalin Dull, Szilárd Póliska, Gyula Ujlaki, Máté Ágoston Demény, Attila Gábor Szöllősi, Borbála Kiss, Andrea Szegedi, Péter Bai, Magdolna Szántó

**Affiliations:** 1 Department of Medical Chemistry, Faculty of Medicine, University of Debrecen, Egyetem ter 1., Elettudomanyi Epulet, H-4032 Debrecen, Hungary; 2 The Hungarian Academy of Sciences, Center of Excellence, Budapest, Hungary; 3 Department of Pathology, Gyula Kenézy Campus, Clinical Centre, University of Debrecen, Debrecen, Hungary; 4 Department of Dermatology, Faculty of Medicine, University of Debrecen, Debrecen, Hungary; 5 Genomic Medicine and Bioinformatics Core Facility, Department of Biochemistry and Molecular Biology, Faculty of Medicine, University of Debrecen, Debrecen, Hungary; 6 Department of Immunology, Faculty of Medicine, University of Debrecen, Debrecen, Hungary; 7 Department of Oncology, Faculty of Medicine, University of Debrecen, Debrecen, Hungary; 8 ELKH-DE Allergology Research Group, University of Debrecen, Debrecen, Hungary; 9 NKFIH-DE Lendület Laboratory of Cellular Metabolism, Debrecen, Hungary; 10 Research Center for Molecular Medicine, Faculty of Medicine, University of Debrecen, Debrecen, Hungary; 11 MTA-DE Cell Biology and Signaling Research Group ELKH, Debrecen, Hungary

**Keywords:** PARP2, Psoriasis, Keratinocyte, Aromatase, Estradiol, NF-κB, PARP inhibitors

## Abstract

**Abstract:**

Poly(ADP-ribose) polymerase 2 (PARP2) alongside PARP1 are responsible for the bulk of cellular PARP activity, and they were first described as DNA repair factors. However, research in past decades implicated PARPs in biological functions as diverse as the regulation of cellular energetics, lipid homeostasis, cell death, and inflammation. PARP activation was described in Th2-mediated inflammatory processes, but studies focused on the role of PARP1, while we have little information on PARP2 in inflammatory regulation. In this study, we assessed the role of PARP2 in a Th17-mediated inflammatory skin condition, psoriasis. We found that PARP2 mRNA expression is increased in human psoriatic lesions. Therefore, we studied the functional consequence of decreased PARP2 expression in murine and cellular human models of psoriasis. We observed that the deletion of PARP2 attenuated the imiquimod-induced psoriasis-like dermatitis in mice. Silencing of PARP2 in human keratinocytes prevented their hyperproliferation, maintained their terminal differentiation, and reduced their production of inflammatory mediators after treatment with psoriasis-mimicking cytokines IL17A and TNFα. Underlying these observations, we found that aromatase was induced in the epidermis of PARP2 knock-out mice and in PARP2-deficient human keratinocytes, and the resulting higher estradiol production suppressed NF-κB activation, and hence, inflammation in keratinocytes. Steroidogenic alterations have previously been described in psoriasis, and we extend these observations by showing that aromatase expression is reduced in psoriatic lesions. Collectively, our data identify PARP2 as a modulator of estrogen biosynthesis by epidermal keratinocytes that may be relevant in Th17 type inflammation.

****Key messages**:**

- PARP2 mRNA expression is increased in lesional skin of psoriasis patients.
- PARP2 deletion in mice attenuated IMQ-induced psoriasis-like dermatitis.
- NF-κB activation is suppressed in PARP2-deficient human keratinocytes.
- Higher estradiol in PARP2-deficient keratinocytes conveys anti-inflammatory effect.

**Supplementary Information:**

The online version contains supplementary material available at 10.1007/s00109-023-02338-z.

## Introduction

Psoriasis is a chronic, immune-mediated, inflammatory skin disorder affecting approximately 2% of worldwide population. The etiology of psoriasis is multifactorial, with genetic and environmental triggers being the most prominent contributors to disease development [1]. To date, psoriasis cannot be cured, hence remains an unmet medical need.

Psoriasis is considered primarily a T cell-mediated disease, where the T helper type (Th)1 and Th17 subsets have central role [2]. The interleukin (IL)23A/IL17A and the tumor necrosis factor (TNF)α/nuclear factor (NF)-κB signaling pathways are considered critical in the pathomechanism of psoriasis [3]. IL23A plays a pivotal role in mediating the expansion and maintenance of IL17-producing Th17 cells [4]. Originally, only IL23A of immune cell origin was believed to be disease relevant, but recently, it was shown that keratinocyte-produced IL23A in itself is sufficient to initiate psoriasis-like skin inflammation [5]. Moreover, the IL17A signaling of keratinocytes has an important contribution to the development of psoriasis in mice [6], suggesting that besides T cells, keratinocytes are also critical players in psoriasis pathogenesis [7]. Keratinocytes are either in a differentiated state or in pathological conditions; they become activated [8]. These two phenotypes are characterized by differential expression of keratins. In healthy epidermis, where terminal differentiation is complete, keratinocytes express keratins 1, 2, and 10, which are crucial for skin barrier integrity. In psoriasis, terminal differentiation is incomplete, and keratinocytes are in a prolonged activated state [8]. Triggers of keratinocyte activation include mostly IL17A and TNFα, as well as interferon (IFN)γ and IL22 [9] that activate NF-κB signaling in keratinocytes [10]. Activated keratinocytes are hyperproliferative and produce pro-inflammatory cytokines and chemokines, including IL1α, IL6, IL8 (CXCL8), and IL23A [5, 11].

PARP enzymes constitute a superfamily of 17 members in humans. Majority of cellular PARP activity is covered by PARP1 (85–90%) and PARP2 (5–15%), which are ubiquitously expressed in mammalian tissues [12]. PARP1 and PARP2 have diverse biological functions ranging from the regulation of DNA repair, cell death, RNA transcription, protein translation, cellular bioenergetics, lipid homeostasis, tumor biology, oxidative stress, and aging [13]. PARP1 and PARP2, despite their structural homology, have distinct biological roles.

PARPs are associated with inflammatory mechanisms in several tissues [14]. The role of PARPs in skin inflammation, however, is a sparsely studied topic, and studies so far focused on PARP1. Genetic deletion or pharmacological inhibition of PARP1 was anti-inflammatory against Th2-mediated inflammation in contact hypersensitivity and irritant dermatitis reactions [15–17], while it was pro-inflammatory in the primarily Th17-mediated imiquimod (IMQ)–induced psoriasis model in mice [18]. Apparently, PARPs have a role in cutaneous inflammation and this role may be dependent on the type of inflammation. The known similarities and differences in the functions of PARP1 and PARP2 prompted us to assess the role of PARP2 in psoriasis.

## Materials and methods

An extended description of materials and methods is given in Supplementary Material.

### CISH

Chromogenic in situ hybridization (CISH) was performed on paraffin embedded skin specimens with double-digoxigenin-labeled PARP2 specific (Custom LNA Detection Probe, Cat. No: 3395005-3DIG, LCD0165598-BKG, Qiagen, Hilden, Germany) or mRNA scramble negative control (Scramble-ISH Custom LNA Detection Probe lncRNA and mRNA, Cat. No: 3395085-3DIG LCD0000002-BDG, Qiagen) locked nucleic acid detection probes according to the manufacturer’s instructions. For visualization alkaline phosphatase conjugated anti-DIG antibody (Roche, Basel, Switzerland) and 4-nitro-blue tetrazolium/5-bromo-4-chloro-indolylphosphate AP chromogen substrate was applied. Slides were counterstained with liquid-stable nuclear fast red.

### Mice

Homozygous PARP2^−/−^ and littermate PARP2^+/+^ male mice on a C57BL/6 J background from heterozygous crossings were used (age 8–12 weeks). The IMQ-induced psoriasis model was performed on mice as in [18]. For aromatase inhibition, 500 μM exemestane solution (in DMSO) was applied on the shaved skin of mice 30 min prior to IMQ treatment. The severity of the lesions, including induration, erythema, and scaling, was scored daily by two experienced, blinded dermatologists on a scale from 0 to 4, where 0 denotes no symptoms and 4 denotes the most severe symptoms.

### Histology

Immunohistochemistry analyses were performed on 4-μm-thick sections cut from formalin fixed, paraffin embedded mouse or human skin samples, similar, as in [19]. Epidermal staining intensities were visually scored on a scale 0–3 by two blinded researchers to quantify histology results. No staining was considered 0, and 3 refers to the maximal intensity seen in the sections.

### Measurement of epidermal thickness

Sections of mouse skin stained with hematoxylin and eosin were examined. The thickness of the epidermal layer was measured using the Fiji ImageJ software. Five points were selected at random on each sections and measured from stratum basale to stratum corneum on vertical sections.

### Cell culture

HPV-Ker cell line was provided by Creative Laboratory Ltd, Szeged, Hungary. HPV-Ker keratinocytes were maintained as in [18]. Post-confluent HPV-Kers were differentiated in culture media supplemented with 1.7 mM Ca^2+^ for 3 days.

### shRNA-mediated gene silencing

PARP2 knocked-down HPV-Ker keratinocytes (shPARP2) were generated by stable transfection with a predesigned shRNA targeting PARP2 (target sequence: ACTATCTGATTCAGCTATTAG; clone ID: TRCN0000235599; ref. sequence: NM_005484) that was cloned into a pLKO.1 vector and packed in lentivirus carrier (Merck KGaA, Darmstadt, Germany). Negative control HPV-Kers (sc) were created by transfection with a non-target shRNA-containing pLKO.1 vector carrying transduction particles (SHC016H; Merck KGaA, Darmstadt, Germany). After transfection, puromycin-resistant cells were selected with 2.5 μg/ml puromycin and subsequently maintained in culture media supplemented with puromycin.

### RNA isolation and RT-qPCR

Total RNA from cells was routinely isolated using TRIzol reagent (TR 118; Molecular Research Center, Inc., Cincinnati, OH, USA) according to the manufacturer’s instructions. For RNA-sequencing, samples were isolated with the RNeasy Kit from Qiagen (Hilden, Germany). RNA samples were treated with Ambion DNase I (AM2222; Thermo Fisher Scientific); afterwards, samples were reverse transcribed using a High-Capacity cDNA Reverse Transcription Kit (4,368,813; Applied Biosystems, Foster City, CA, USA,). The RT-qPCR reactions were performed in a Light-Cycler 480 Detection System (Roche Applied Science, Basel, Switzerland) using TaqMan assays (listed in Supplementary Material).

### RNA-seq

High throughput mRNA sequencing analysis was performed on Illumina sequencing platform. RNA sample quality was checked on Agilent BioAnalyzer using Eukaryotic Total RNA Nano Kit according to manufacturer’s protocol. RNA-Seq libraries were prepared from total RNA using Ultra II RNA Sample Prep kit (New England BioLabs) according to the manufacturer’s protocol. Moderated *t*-test with Benjamini–Hochberg FDR was used to determine differentially expressed genes between conditions. The CytoScape v3.4 software with ClueGo v2.3.5. application was used for identifying over-represented Gene ontology (GO) terms. Two-sided hypergeometric test with Bonferroni step down correction was performed using the list of differentially expressed genes and GO Biological process database.

### In silico analysis of RNA-seq data

In silico analysis was performed on transcriptomics data from healthy skin and lesional skin of psoriasis patients already published by other groups (PubMed Unique Identifier (PMID): 24,441,097 [20]) and our own RNASeq data. Raw read counts from both sources were standardized, and heatmaps were generated using GraphPad Prism 9.1.2.

### Protein extraction and Western blotting

Whole-cell or nuclear protein samples were isolated and further processed for SDS-PAGE/Western blotting as detailed in [21]. Antibodies used in blots are listed in Supplementary Material.

### Cell cycle analysis of keratinocytes

Cells were fixed with ice-cold 70% ethanol. Cells were washed with staining buffer; then, 100 µg/ml propidium iodide stock solution (Invitrogen Corporation, Carlsbad, CA, USA, V13242B) was added to the samples (1 µl/sample) and incubated for 20 min in dark. Cell cycle analysis was carried out by NovoCyte Flow Cytometer (ACEA Biosciences, Inc, Agilent, Santa Clara, CA, USA).

### Mouse cytokine array

Mouse skin samples were flash frozen in liquid nitrogen after excise. Tissues were homogenized in a TissueLyser II device (Qiagen, Hilden, Germany). Supernatants were used for the cytokine array according to the manufacturer’s protocol (Proteome Profiler Array, No. ARY006, R&D Systems/Bio-Techne, Minneapolis, MN, USA). The immunoblot images were analyzed using the CellProfiler for non-cell images analysis software (Broad Institute of MIT and Harvard, Cambridge, MA, USA) as in [22].

### Determination of cytokine and estradiol concentration

Supernatants were collected from HPV-Kers, and mouse skin protein samples were analyzed for cytokines and estradiol using commercially available ELISA kits according to the manufacturers’ protocols. The ELISAs used in this study are listed in Supplementary Material.

### Immunocytochemistry

Confocal microscopy was performed as in [21]. Antibodies used are listed in Supplementary Material.

### NF-κB activity assay

Protein was extracted from the cells using Complete Lysis Buffer AM2 (provided from TransAM™ NFκB p65 Chemi Kits, 40,097). NF-κB p65 subunit activation was determined in 4 µg of whole-cell protein extracts from each sample using the TransAM™ NFκB p65 Chemi Kits (Active Motif, Carlsbad, CA, USA, 40,097) according to the manufacturer’s instructions. Luminescence was measured using a Spark 10 M microplate reader (Tecan, Mannedorf, Switzerland).

### Statistics

The distribution of data was analyzed by Shapiro–Wilk test. If two groups were compared, we used independent *t*-test (two-tailed), as the Shapiro–Wilk test showed normal distribution. If the distribution was not normal, Mann–Whitney test was used. When we compared more than two groups and if the distribution was normal, we used ANOVA followed by Tukey’s post hoc test. In case the data did not show a normal distribution, Kruskal–Wallis test were applied complemented by Dunn’s post hoc test. Intensity of IHC reactions was scored on a scale 0–3, and subsequently, *χ*^2^-test was applied for statistical analysis between the groups. The significance level was set at 0.05.

## Results

### PARP2 expression is increased in psoriasis and PARP2 deletion decreases the severity of IMQ-induced psoriasiform dermatitis in mice

**Fig. 1:**
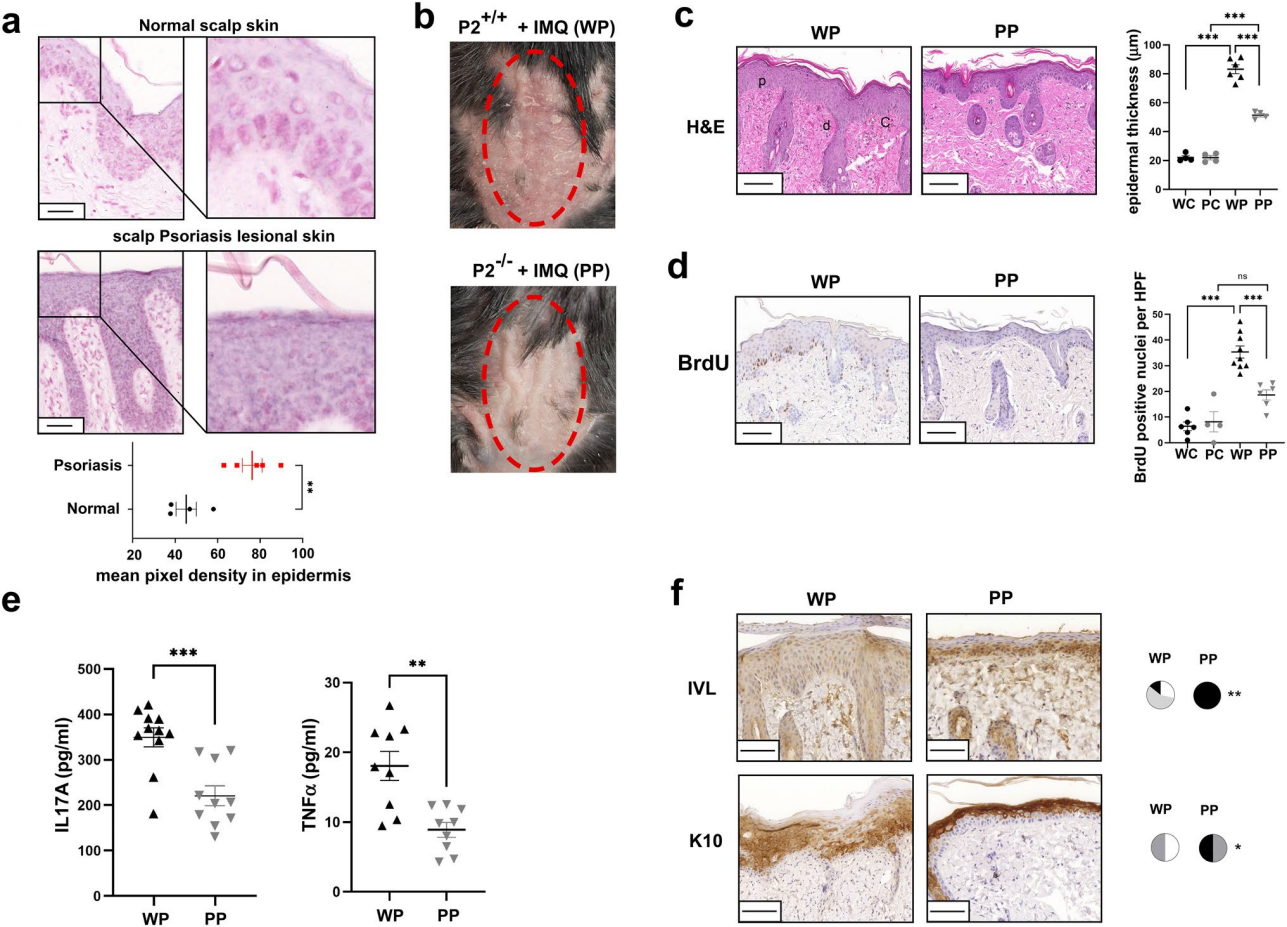
PARP2 expression is increased in psoriasis, and PARP2 deficiency has a protective role in the IMQ-induced model of psoriasis. **a** CISH was performed on psoriatic lesions and on normal skin of healthy controls (*n* = 5/4). Both lesional and healthy samples were localized to the scalp region of subjects. Right images are magnified sections of the pictures on the left. Scale bars: 50 μm. Purple dots indicate PARP2 mRNA expression. Pixel density in the blue color channel was measured by Fiji ImageJ software. **b** Psoriasiform dermatitis was induced in PARP2^+/+^ and PARP2^−/−^ mice by the IMQ-containing Aldara cream on the shaved back skin of mice. Pictures show the lesions on day 5. **c** Hematoxylin and eosin (H&E) staining of lesional sections of mice, and characteristics of IMQ-induced dermatitis are highlighted as p = proliferating basal cells, c = dilated capillaries, d = dermal cellular infiltrate. Epidermal thickness in lesions of mice was measured by the Fiji ImageJ software, and depicted as mean of *n* = 4–10 per group ± SEM. **d** Detection of BrdU incorporation was used to determine proliferation of epidermal keratinocytes. Number of BrdU positive nuclei was counted in three high power fields (HPF) in each sections of study mice, and the average number per HPF per mouse is shown as mean of *n* = 4–10 per group ± SEM. **e** IL17A and TNFα ELISAs were performed from whole-skin lysates of mice. **f** Pictures show involucrin (IVL) and keratin (K)10 IHC performed on lesional skin of mice. WC denotes wild-type (PARP2^+/+^) vehicle-treated mice, WP denotes wild-type (PARP2^+/+^) mice with IMQ-induced psoriasis-like dermatitis, PC denotes PARP2^−/−^ vehicle-treated mice, PP denotes PARP2^−/−^ mice with IMQ-induced psoriasis-like dermatitis. Scale bars: 100 μm. Statistical analysis was performed by GraphPad Prism. In panels **c** and **d**, as the Shapiro–Wilk test showed normal distribution, one-way ANOVA followed by Tukey’s multiple comparisons test; in panel **a** and **e** unpaired, two-tailed *t*-tests were used for determination of statistical significance. Intensity of IHC reactions was scored on a scale 0–3, and subsequently, *χ*^2^-test was applied for statistical analysis between the groups. *p < 0.05; **p < 0.01; ***p < 0.001

We assessed the expression of PARP2 in lesional skin biopsies of psoriasis patients and in normal skin from similar regions of healthy subjects. We found low basal expression of PARP2 mRNA in normal skin, but psoriatic epidermis displayed strong induction of PARP2 mRNA expression (Fig. 1a). To characterize the functional significance of PARP2 expression in psoriasis, we applied an acknowledged murine model of psoriasis, using imiquimod (IMQ)-induced dermatitis on PARP2^−/−^ and littermate PARP2^+/+^ male mice. Macroscopically, the developed lesions were less severe in the IMQ-treated PARP2^−/−^ (PP) mice than in IMQ-treated wild-type (WP) mice (Fig. 1b). Microscopically, hematoxylin and eosin staining revealed hallmarks of IMQ-induced dermatitis, such as increased keratinocyte proliferation in the basal layer, dilated capillaries, and dermal cellular infiltration in lesional skin sections of WP mice, but to a lesser extent in the sections of PP mice (Fig. 1c). Thickening of the epidermis was more pronounced in the skin of WP mice than in PP mice as compared to vehicle-treated wild-type (WC) and PARP2^−/−^ (PC) mice (Fig. 1c). In good agreement, markedly fewer BrdU positive nuclei were visible in the epidermis of PP mice compared to that of WP mice (Fig. 1d), suggesting a slower proliferation rate of PARP2^−/−^ keratinocytes. Attenuated inflammation in PP mice is evidenced by the lower level of IL17A and TNFα in the lesional skin lysates of PP mice compared to WP mice (Fig. 1e). Immunohistochemical analyses of lesional sections show that the early differentiation marker involucrin maintained its typical expression in stratum spinosum in PP mice, while in the WP group, involucrin spread across the epidermis (Fig. 1f). In addition, the late terminal differentiation marker keratin 10 displayed a significantly higher expression in the epidermis of PP mice than in WP mice (Fig. 1f). Taken together, these data suggest a role for PARP2 in keratinocyte activation associated with psoriatic inflammation.

### Terminal differentiation of PARP2-depleted human keratinocytes remains intact upon treatment with psoriasis-related cytokines

**Fig. 2:**
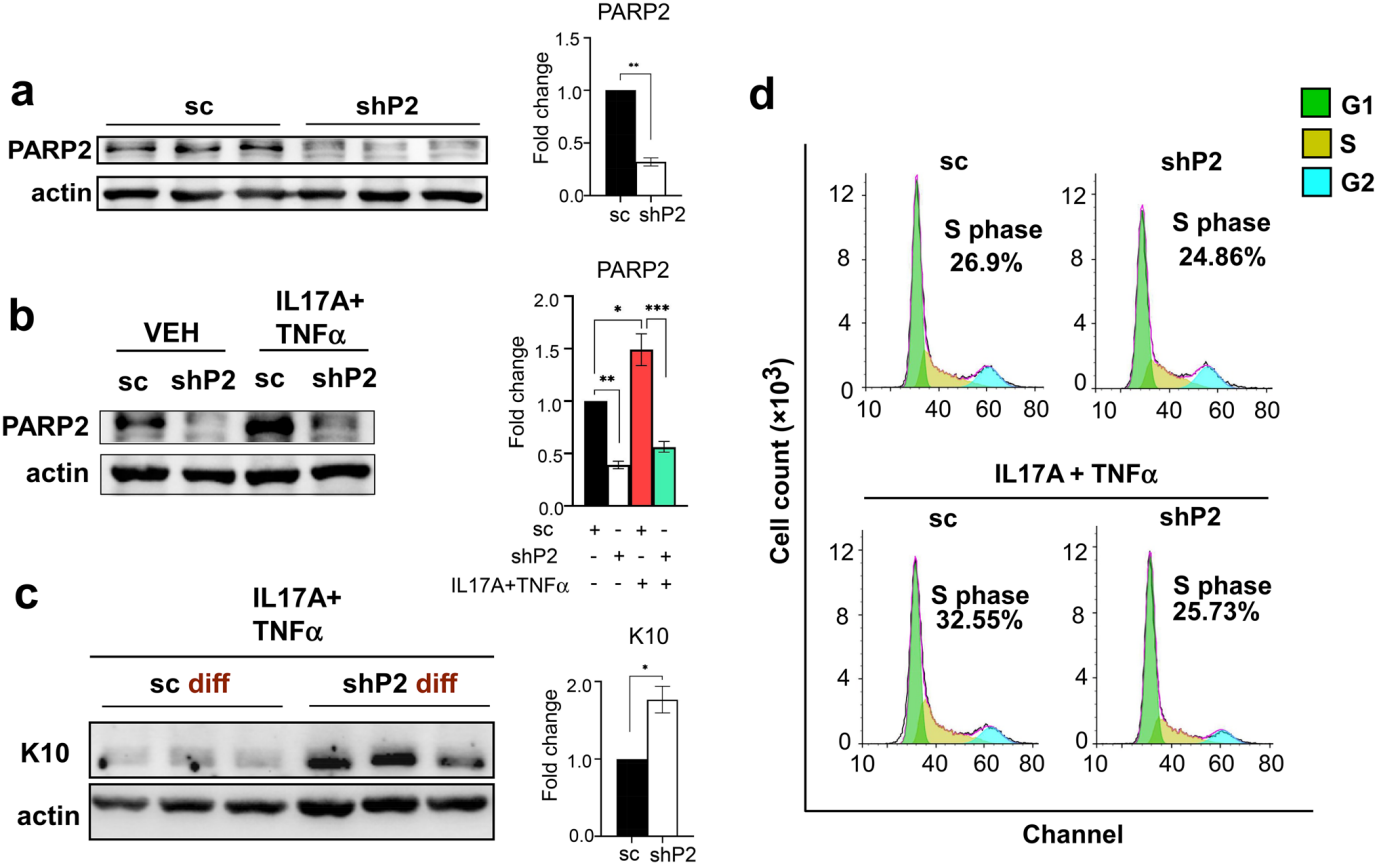
PARP2 knock-down in human keratinocytes copies the antipsoriatic phenotype observed in PARP2^−/−^ mice. **a** HPV-Ker human keratinocytes were transduced by either a negative control sequence- or a PARP2-specific shRNA-containing lentivirus giving rise to sc control and PARP2-silenced shP2 keratinocytes. **b** sc and shP2 HPV-Kers were treated with either vehicle (VEH) or the combination of 200 ng/ml IL17A and 10 ng/ml TNFα for 6 h, and afterwards, PARP2 protein was detected in the samples. **c** Post-confluent sc and shP2 HPV-Kers were differentiated in the presence of IL17A and TNFα and, subsequently, K10 was determined by immunoblotting. **d** Cell cycle analysis of sc and shP2 cells upon treatment with either vehicle or IL17A + TNFα. Histograms and blots are representatives of three independent experiments. Optical densities (O.D.) of blots were determined in the ImageLab software. Mean O.D. values are presented as the ratio of O.D. to actin, relative to that in sc cells as fold change ± SEM

To characterize the role of PARP2 specifically in keratinocytes, PARP2 was knocked-down in HPV-Ker human keratinocytes by the application of a PARP2-specific short hairpin (sh)RNA-containing lentiviral vector that resulted in approximately 70% decrease in PARP2 protein expression in shP2 HPV-Ker cells compared to the negative control sequence-bearing sc cells (Fig. 2a). We stimulated sc and shP2 HPV-Kers with the combination of IL17A and TNFα, as it was previously established that the synergistic action of IL17A and TNFα on keratinocytes induces such transcriptomic changes that show a massive correlation with the psoriasis gene signature [23]. Treatment with IL17A and TNFα resulted in elevated PARP2 expression in keratinocytes (Fig. 2b). When differentiated in the presence of IL17A and TNFα, shP2 keratinocytes displayed markedly higher expression of CK10 than the sc keratinocytes (Fig. 2c). In cell cycle analyses, we detected an increase in the proportion of S-phase cells in sc HPV-Kers, but not in shP2 cells, upon treatment with IL17A + TNFα (Fig. 2d). These results indicate that the absence of PARP2 in keratinocytes has a protective effect against IL17A-evoked stimulation of proliferation and inhibition of differentiation that are key events in the pathomechanism of psoriasis [24]. Apparently, PARP2-depleted human keratinocytes phenocopied the role of PARP2 in human psoriatic skin and in a murine psoriasis model, which called for further investigations into the role of PARP2 in inflammatory regulation in keratinocytes.

### Suppression of pro-inflammatory cytokine-mediated NF-κB activation in PARP2-deficient keratinocytes

**Fig. 3:**
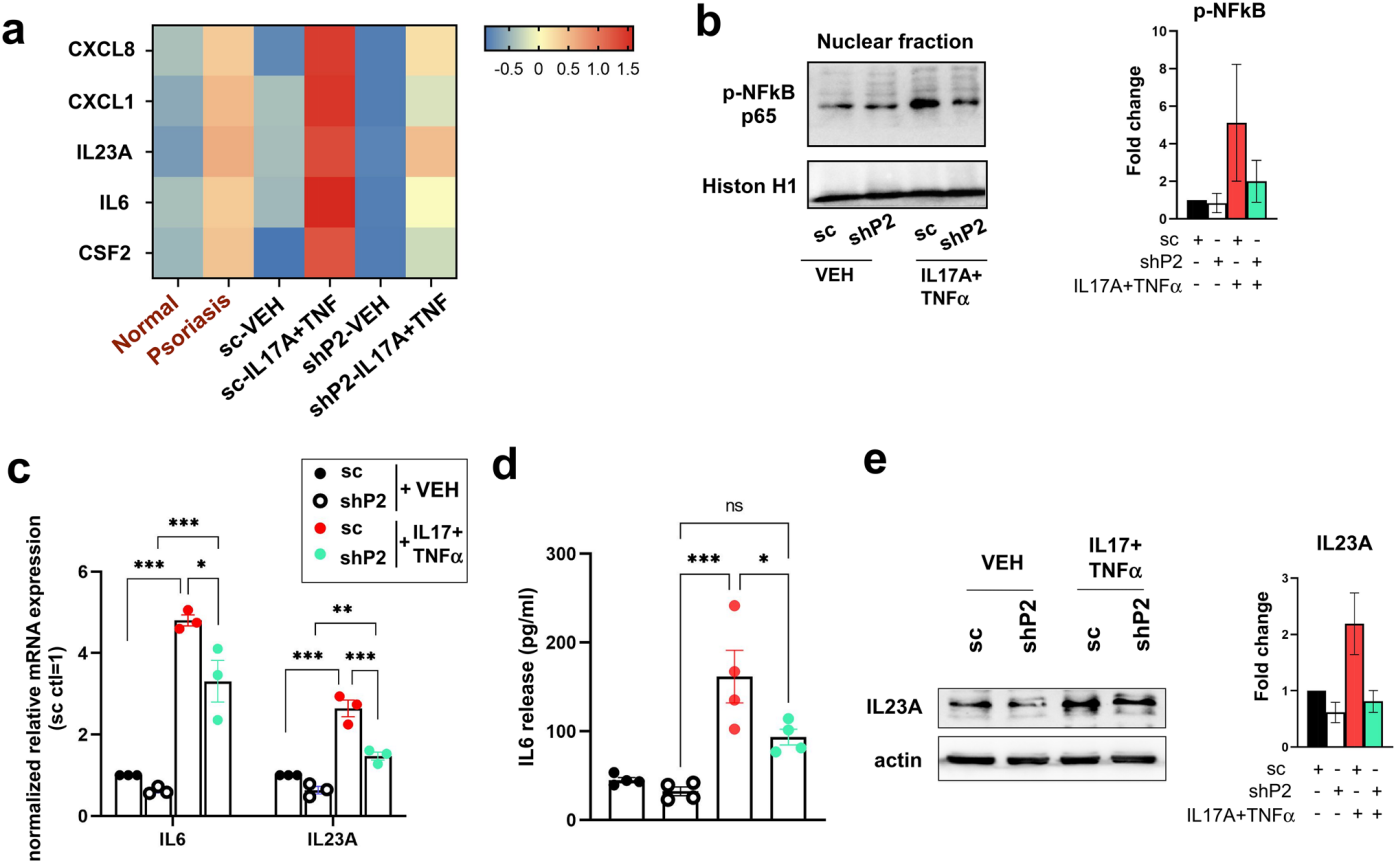
PARP2 silencing inhibits activation of NF-κB. **a** Heatmaps were generated from publicly available RNA-seq data of healthy skin and skin of psoriasis patients, as well as from our RNA-seq data of VEH or IL17A + TNFα-treated sc and shP2 HPV-Kers, and show expressions of a set of NF-κB target genes. **b** Phosphorylation of the p65 subunit of NF-κB was detected in the nuclear fraction of sc and shP2 HPV-Kers treated with either vehicle or IL17A + TNFα. **c** IL6 and IL23A mRNA expression was measured by RT-qPCR. IL6 and IL23A expressions were normalized to the expression of GAPDH, and are expressed relative to the expressions measured in sc VEH control cells. Data are depicted as the mean of three independent measurements ± SEM. **d** Measurement of IL6 secretion from vehicle and IL17A + TNFα- treated sc and shP2 keratinocytes was performed by ELISA. Data depicts the results of four biological replicates as mean ± SEM. **e** Immunoblot shows IL23A protein expression in sc and shP2 HPV-Kers after stimulus with vehicle or IL17A + TNFα. Blots are representatives of at least two independent experiments. O.D. of blots were determined in the ImageLab software. Mean O.D. values are presented as the ratio of O.D. to actin, relative to that in VEH-treated sc cells as fold change ± SEM. For panels **c**, **d**, and **e**, the comparison between groups was made by one-way ANOVA followed by Tukey’s multiple comparisons test. *p < 0.05, **p < 0.01, ***p < 0.001

To find out about the PARP2-regulated mechanisms in keratinocytes, we performed RNA-seq analyses from sc and shP2 HPV-Kers treated with either vehicle or IL17A + TNFα. We identified over 300 differentially expressed genes between vehicle-treated sc and shP2 keratinocytes. When comparing vehicle versus IL17A + TNFα-treated sc HPV-Kers, we found 64 differentially expressed genes in contrast to only 20 between vehicle versus IL17A + TNFα-treated shP2 cells, suggesting the suppression of IL17A and TNFα-induced pathways in PARP2-deficient keratinocytes. TNFα alone, or in concert with IL17A, acts mainly through the activation of NF-κB [25], a critical pro-inflammatory pathway in the development of psoriasis. Indeed, we performed in silico analysis of already published RNA-seq data from 92 psoriatic and 82 normal skin samples [20], and we found that several NF-κB target genes, including CXCL8, chemokine ligand 1 (CXCL1), IL23A, IL6, and colony stimulating factor (CSF)2, are upregulated in psoriatic skin (Fig. 3a). In our RNA-seq data, we found an even higher upregulation of these genes in sc HPV-Kers as a result of IL17A + TNFα treatment, but much lower induction was seen in shP2 cells (Fig. 3a).

Confirming these data, the level of phosphorylated p65 increased in the nuclear fraction of sc HPV-Kers, but not in shP2 cells, after stimulation with IL17A and TNFα (Fig. 3b). We chose two markers for further evaluations, IL6 and IL23A that were both associated with psoriatic inflammation in humans and in murine models [5, 11, 26]. Validating the RNA-seq data RT-qPCR measurements showed that IL17A and TNFα triggered a significantly less elevation of IL6 and IL23A mRNA expression in shP2 keratinocytes than in sc keratinocytes (Fig. 3c). In addition, IL17A + TNFα-induced IL6 secretion (Fig. 3d) and IL23A protein expression (Fig. 3e) was lower in shP2 keratinocytes than in the sc cells. These data suggest that PARP2 may modulate NF-κB activity and hence NF-κB-mediated inflammatory pathways.

### Increased estradiol production suppresses NF-κB in PARP2 depleted keratinocytes

**Fig. 4:**
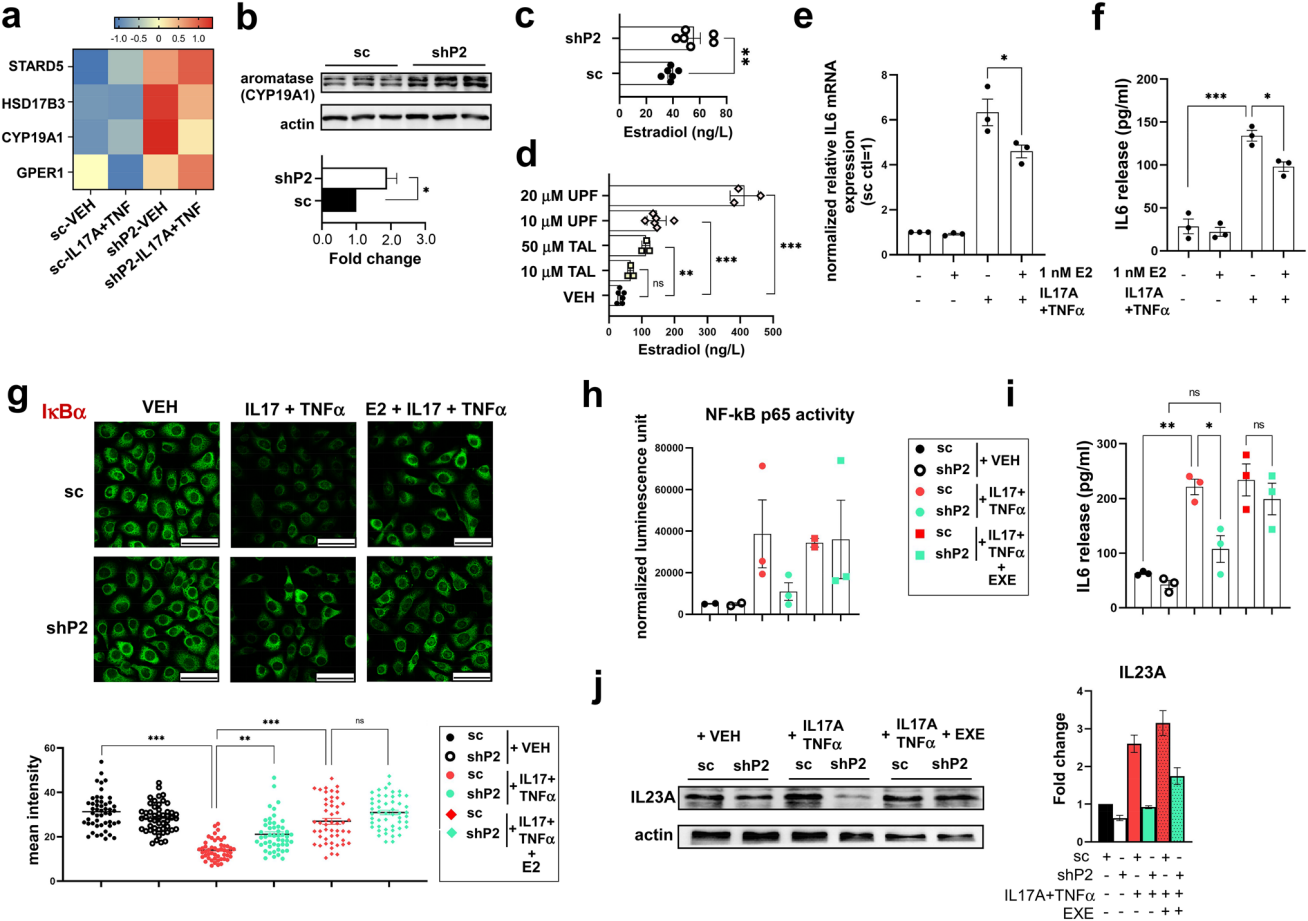
Higher estradiol secretion in PARP2-deficient keratinocytes is anti-inflammatory. **a** Heatmaps show expression of genes involved in the synthesis and action of estrogens in vehicle (VEH) or IL17A + TNFα-treated sc and shP2 HPV-Kers. The average of expressions measured in three biological replicates are depicted. **b** Aromatase expression was determined in sc and shP2 keratinocytes by immunoblotting. **c** ELISA measurement of estradiol (E2) secretion in the supernatants of sc and shP2 HPV-Kers, and **d** HPV-Kers treated with either VEH or the indicated concentrations of talazoparib (TAL) or UPF1069 (UPF) for 48 h. Data is depicted as the mean of at least three biological replicates ± SEM. **e** and **f** Culture media of HPV-Kers was supplemented with 1 nM estradiol prior to treatment with either vehicle or IL17A + TNFα. Subsequently, IL6 mRNA expression was assessed in cell extracts by RT-qPCR (**e**), and the supernatants of cells was used for measuring IL6 production by ELISA (**f**). Data represents the mean of three independent measurements ± SEM. **g** Immunofluorescence shows IκBα in sc and shP2 in the indicated treatments. Scale bars: 50 μm. Data presented are representative of two independent experiments. Fluorescence intensity was measured in 50 cells per condition, and the mean intensity ± SEM is depicted. **h** Activity of p65 and **i** IL6 concentration was measured in sc and shP2 HPV-Kers upon treatment with either vehicle, or IL17A + TNFα, or upon the application of 5 μm exemestane (EXE) prior to treatment with IL17A + TNFα. Graphs show the mean of at least two independent experiments ± SEM. **j** IL23A protein expression was determined by immunoblotting. O.D. of blots were determined in the ImageLab software. Mean O.D. values are presented as the ratio of O.D. to actin, relative to that in VEH-treated sc cells as fold change ± SEM from three independent experiments. For the comparison of groups, we used unpaired, two-tailed *t*-test in panels **b** and **c**. In panels **d**, **e**, **f**, and **i**, we used one-way ANOVA followed by Tukey’s multiple comparisons test as the Shapiro–Wilk test showed normal distribution of data. In panels **g** and **j**, Kruskal–Wallis test was applied. Significance was set at *p < 0.05, **p < 0.01, or ***p < 0.001

When mining the RNA-seq dataset, we found markers of steroidogenesis (such as the cholesterol transporter StarD5) that were induced in shP2 HPV-Kers compared to their sc counterparts (Fig. 4a), which was in agreement with our prior studies linking PARP2 to cholesterol and steroid homeostasis [21, 27]. We found increased expression of HSD17B3, which encodes 17β-Hydroxysteroid dehydrogenase 3 that catalyzes the conversion of androstenedione to testosterone, and CYP19A1, which encodes aromatase that converts testosterone to estrogens. In addition, G protein-coupled estrogen receptor 1 (GPER1) expression was induced in shP2 cells (Fig. 4a), suggesting increased estrogen response. These gene expression alterations caught our interest since estradiol [28, 29], the major human estrogen, and GPER1 [30] are both known to be able to suppress NF-κB activation in several tissues. Therefore, we hypothesized that inhibition of NF-κB activation may be a consequence of the induction of estrogen action in shP2 cells.

First, we confirmed that silencing of PARP2 induced the expression of aromatase in keratinocytes (Fig. 4b). In accordance, we detected a significantly higher concentration of estradiol in the supernatants of shP2 HPV-Kers than in those of sc HPV-Kers (Fig. 4c). Next we turned to assess whether the enzymatic activity of PARP2 could play a role in upregulating estradiol production. To that end, we inhibited PARP2 with a specific inhibitor UPF1069 that induced a robust increase in estradiol secretion of HPV-Kers (Fig. 4d). Moreover, the application of the pan-PARP inhibitor talazoparib, a potent inhibitor of both PARP1 and PARP2, led to a modest induction of estradiol production in HPV-Kers compared to UPF1069 (Fig. 4d), which confirms that the enzymatic activity of PARP2 is involved in estradiol regulation in keratinocytes.

When HPV-Kers were kept in estradiol containing media prior to stimulation with IL17A and TNFα, lower IL6 mRNA expression (Fig. 4e) and secretion (Fig. 4f) were measured, indicating that estradiol is indeed anti-inflammatory against IL17A + TNFα-induced response in keratinocytes.

We detected IL17A + TNFα-induced degradation of inhibitor of nuclear factor kappa B alpha (IκBα) that retains NF-κB in cytoplasm in sc HPV-Kers that was less pronounced in shP2 cells (Fig. 4g). However, when sc HPV-Kers received estradiol in advance of the cytokine stimulus, IκBα degradation was hindered (Fig. 4g), which corroborates with previous findings showing estradiol-mediated inhibition of IκBα degradation [29].

To functionally link estrogen action to the antipsoriatic phenotype of PARP2 silencing, estradiol synthesis was inhibited in HPV-Kers by the application of the aromatase inhibitor exemestane. The activation of p65 in response to IL17A and TNFα increased in shP2 cells to the level measured in sc cells upon exemestane treatment (Fig. 4h). In agreement, exemestane application blunted the difference in IL17A + TNFα-induced IL6 secretion (Fig. 4i) and IL23A protein expression (Fig. 4j) between sc and shP2 HPV-Kers. Apparently, inhibition of estradiol synthesis with exemestane abolished the suppression of NF-κB activation in shP2 keratinocytes, suggesting that the anti-inflammatory effect of PARP2-silencing in keratinocytes stemmed from increased estradiol production.

#### Aromatase function is required for PARP2-mediated anti-inflammatory effect in murine skin

We studied the effect of cutaneous aromatase inhibition on the IMQ-induced psoriasis in mice. We performed the IMQ-induced dermatitis as follows: PARP2^+/+^ and PARP2^−/−^ mice that were treated with vehicle and IMQ (WP and PP mice, respectively), and PARP2^+/+^ and PARP2^−/−^ mice that were treated with topical exemestane solution prior to treatment with IMQ (WPE and PPE mice, respectively).

**Fig. 5:**
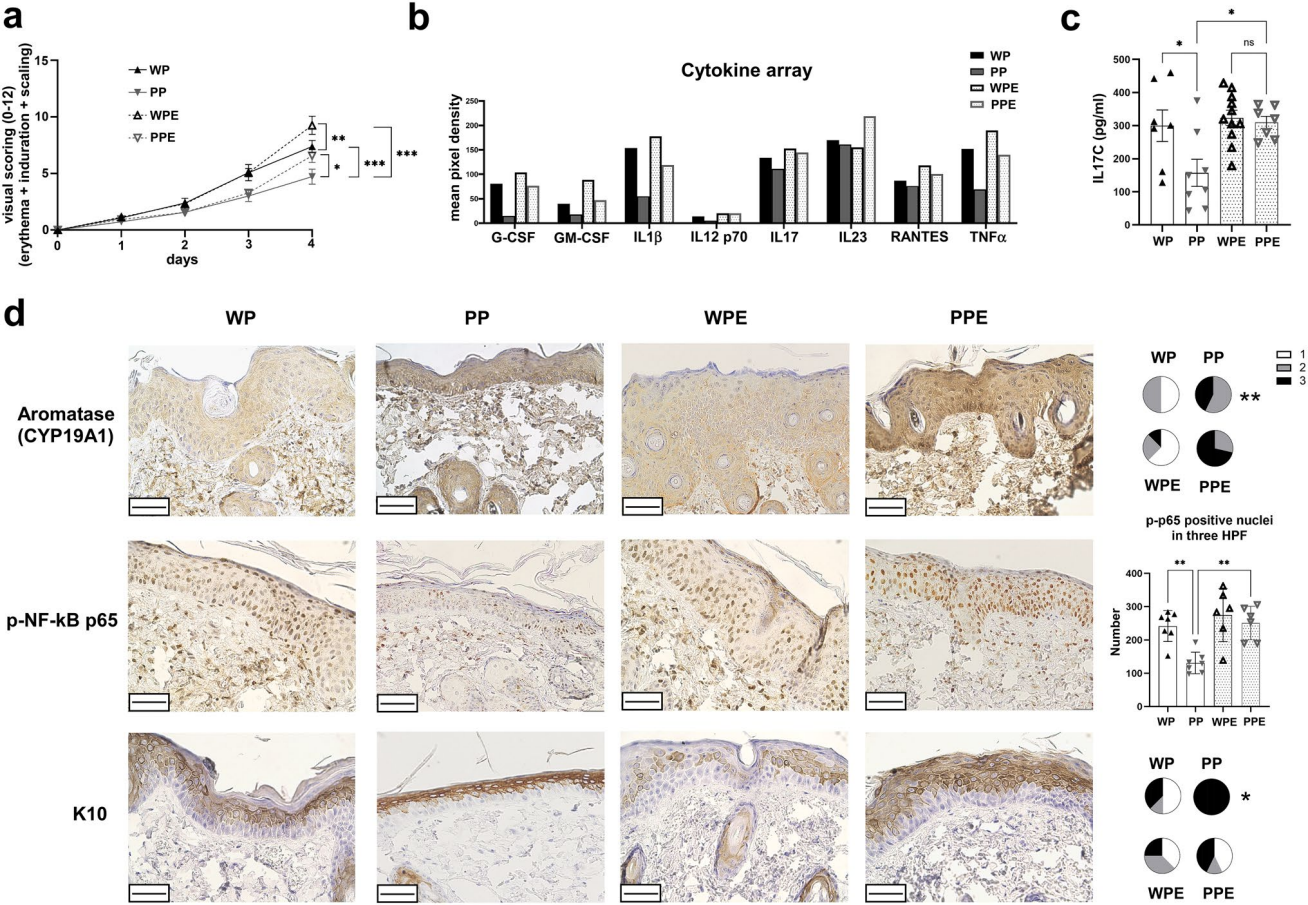
Exemestane abolishes the protective effect of PARP2 deletion in IMQ-induced psoriasis. **a** Shaved back skin of PARP2^+/+^ and PARP2^−/−^ mice were treated with either vehicle plus IMQ (WP and PP mice) or 500 μM exemestane (EXE) plus IMQ (giving rise to WPE and PPE mice), and the developed dermatitis was scored as follows: erythema, epidermal induration, and scaling (each on a scale 0–4). The cumulative score is presented on a scale 0–12. For comparison of groups (*n* = 7–11 mice/ group), we used two-way ANOVA followed by Tukey’s multiple comparisons test. **b** Cytokine array was used to determine pro-inflammatory and psoriasis-related cytokines in whole-skin lysates of mice of WP, PP, WPE, and PPE groups. Data represents the mean pixel density of the developed dots from pooled samples corresponding to the listed cytokines on the array membranes. **c** IL17C was determined in each skin lysates by ELISA. **d** IHC was used for detection of aromatase, phosphorylated p65 NF-κB subunit, and CK10 in the epidermis. Scale bars: 50 μm. Intensity of CK10 and aromatase IHC reactions was scored on a scale 0–3, and subsequently, *χ*^2^-test was applied for statistical analysis between the groups. For the evaluation of p65 phosphorylation, positive nuclei were counted in three high-power fields (HPF) in each sections, and the average number per HPF is shown. For the comparison of groups in p65 phosphorylation, we used one-way ANOVA followed by Tukey’s multiple comparisons test. *p < 0.05, **p < 0.01, ***p < 0.001

Exemestane treatment significantly increased the cumulative visual score of the IMQ-induced lesions in case of both PARP2^+/+^ and PARP2^−/−^ mice (Fig. 5a), indicating that aromatase might have a protective role in the skin. Exemestane abolished the anti-inflammatory effect of PARP2 deletion in the IMQ model as determined by the concentration of several psoriasis-related cytokines measured in the skin of mice (Fig. 5b). IL17C, a key cytokine in psoriatic inflammation [31], was measured separately, and showed a similar pattern within the four groups of mice to the other cytokines in the array (Fig. 5c).

Immunohistochemistry of lesional skin sections showed higher epidermal aromatase expression in PP and PPE mice compared to WP and WPE mice, recapitulating our findings in human keratinocytes. Concordantly, WP mice displayed a significantly higher number of phosphorylated p65 positive nuclei in the epidermis compared to PP mice (Fig. 5d). However, aromatase inhibition resulted in increased p65 phosphorylation, which was more pronounced when comparing PPE mice compared to PP mice, but also tended to increase in WPE mice compared to WP mice (Fig. 5d). In addition, aromatase inhibition negatively affected keratinocyte differentiation as determined by decreased CK10 reactivity in the epidermis of exemestane-treated mice compared to those treated with solely IMQ (Fig. 5d).

Taken together, these results imply that the protection against the severity of IMQ-induced dermatitis seen in PARP2^−/−^ mice was, at least in part, a consequence of higher epidermal aromatase activity.

### Aromatase expression is decreased in psoriatic skin

**Fig. 6:**
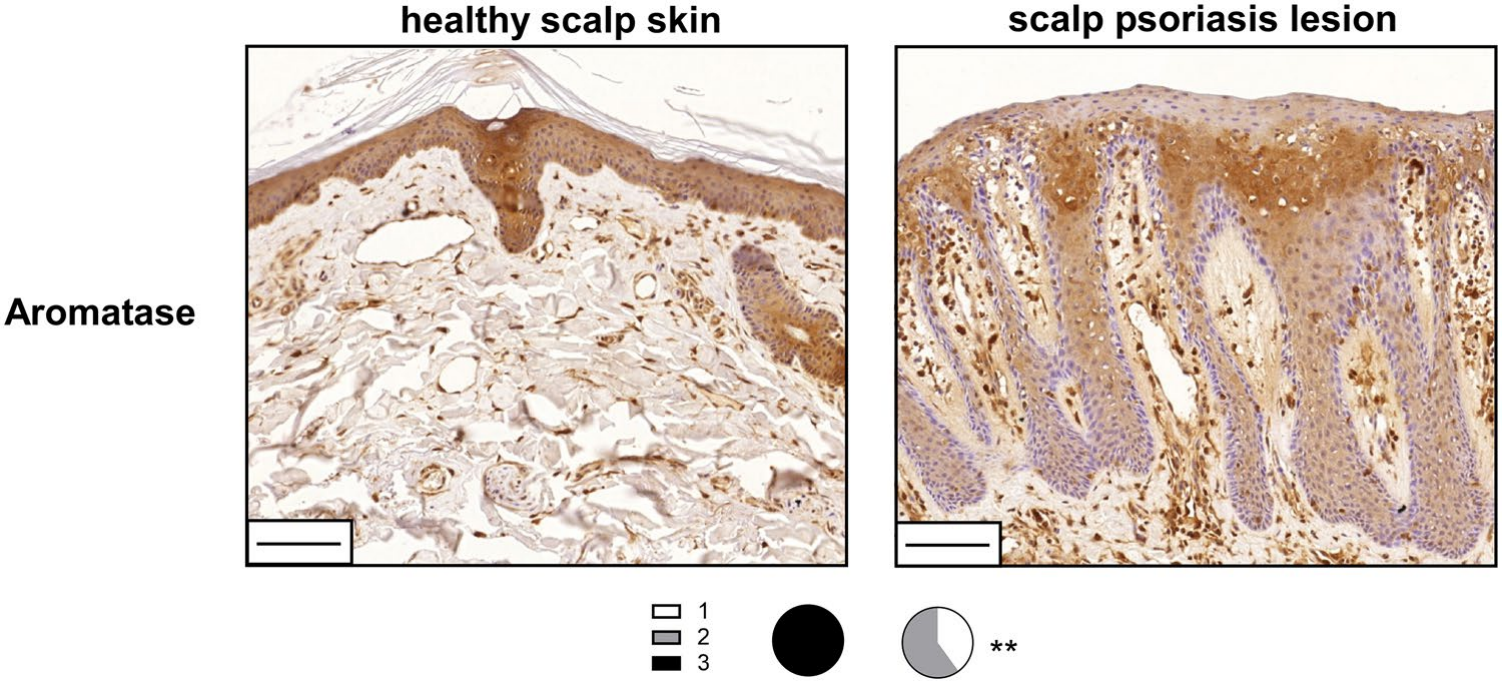
Decreased aromatase expression in psoriatic lesions. Aromatase IHC was performed on lesional skin sections of psoriasis patients and healthy skin of control subjects (*n* = 5/5). Both psoriatic lesions and healthy samples were localized to the scalp region of subjects. Scale bars:100 μm. Intensity of IHC reactions was scored on a scale 0–3, and subsequently *χ*^2^-test was applied for statistical analysis between the groups. **p < 0.01

Finally, we investigated aromatase expression in human skin using the same psoriatic and healthy samples in which we analyzed PARP2 expression. In healthy samples, we found high expression of aromatase that evenly spread across the epidermis, while in psoriatic epidermis, aromatase expression displayed an overall reduction and was restricted to the stratum granulosum (Fig. 6), which indicates that estrogen biosynthesis of the skin is suppressed in psoriasis. This observation together with the above findings suggests that there may be an interconnectedness between PARP2 expression and estrogen production in epidermal keratinocytes that may contribute to the fine-tuning of local inflammation during psoriasis pathomechanism.

## Discussion

The skin has the ability to synthesize steroid hormones that may exert autocrine or paracrine effects. These locally produced glucocorticoids, estrogens, and androgens affect epidermal homeostasis and local immune functions, which suggests association between disrupted cutaneous steroid biosynthesis and skin inflammation [32]. Prior art already suggested links between changes in endogenous steroid biosynthesis and psoriasis. Namely, glucocorticoid production is suppressed in psoriatic skin [33]. Furthermore, the course of psoriasis changes in women during pregnancy, postpartum, and with menopause, which indicates that estrogen may influence psoriasis [34]. Still, little is known about the regulation of local steroidogenesis in the skin and its implication in psoriasis development.

PARP2 has been associated with several aspects of steroid synthesis and steroid mechanism of actions mostly in metabolic tissues [35]. In skeletal muscle of PARP2^−/−^, mice–induced expression was found of 17β-dehydrogenase 11 (HSD17B11), an enzyme involved in androsterone biosynthesis, and 5α-reductases (SRD5A1, 2) that catalyze the conversion of testosterone to dihydrotestosterone (DHT) [21]. As a consequence, muscular levels of DHT increased in the skeletal muscle of PARP2^−/−^ mice, without changing systemic levels of DHT [21]. In addition, PARP2 was identified as a contributor to androgen receptor signaling in prostate [36]. In this study, we demonstrate that the genetic deletion or depletion of PARP2 has a protective effect against psoriasis-like inflammation in the IMQ-induced murine model and in human keratinocyte cultures and that this protection is dependent on aromatase function and estradiol synthesis, and estradiol-mediated suppression of NF-κB activity in keratinocytes.

The therapeutic potential of estrogens in psoriasis has been suggested [34, 37], but long-term systemic estrogen therapy is not considered suitable due to negative side effects, and exogenous estrogen treatment has never been attempted in psoriasis. With our results, we propose the stimulation of keratinocytes’ innate estrogen production by targeting PARP2 as a potential approach in psoriasis management. However, several questions remain unanswered in our study that may raise concerns with the feasibility of this concept.

In fact, the role of estrogens in immune modulation is not straightforward. Although psoriasis tends to improve during pregnancy and exacerbate in menopause, a minority of patients experienced worsening of symptoms during pregnancy [38, 39]. The positive effects of estrogens on psoriasis may be explained by the apparent ability of estrogens to create a shift from a primarily Th1 and Th17-mediated immunity to a primarily Th2-mediated immunity [37]. In contrast to that, the activity of estrogen receptors (ER) display profound dose and cell type dependency, and therefore, ER activity may lead to either induction or suppression of pro-inflammatory cytokine production as a function of estrogen concentrations [40]. The estradiol level necessary for the inhibition of NF-κB was actually determined in human cells [41], and according to the study, the minimum concentration of estradiol needed to achieve anti-inflammatory effect is somewhere in the 10^−10^ M range, which approximately corresponds to the concentration we detected in the supernatants of sc and shP2 HPV-Kers. It is therefore possible that subtle increases in estradiol production may suffice to turn on the anti-inflammatory machinery in keratinocytes, such as in the case of PARP2-silenced cells. The estradiol level in the skin may show strong intrapersonal and interpersonal variability that requires further investigations to determine the potentially therapeutic concentration of estrogens in psoriasis.

PARP2 may also have a complex role in inflammatory regulation. PARP2 deletion does not seem to influence Th2-mediated inflammatory processes, whereas the deletion or inhibition of PARP1 was anti-inflammatory in mouse models of contact hypersensitivity reaction [15–17], asthma [42], acute pancreatitis [43], allergic airway inflammation [44], and experimental colitis [45]. In sharp contrast, we previously reported exacerbation of the Th17-mediated IMQ-induced psoriasis-like dermatitis in PARP1^−/−^ mice [18], while the present study shows the beneficial effect of PARP2 deletion in the IMQ model. Of note, the IMQ-induced psoriasis model is not the first Th17-mediated process where PARP1^−/−^ and PARP2^−/−^ mice displayed opposing phenotypes. In experimental autoimmune encephalomyelitis (EAE), a murine model of multiple sclerosis, PARP1 deletion increased [46], while PARP2 deletion reduced EAE-associated neuroinflammation [47]. Interestingly, multiple studies reported that estrogens protect from neuroinflammation in EAE [48–50] that aligns well with our findings. We might as well assume that PARP2 is related to estrogen action in Th17-mediated inflammatory processes. Based on our findings, we hypothesize that during the progression of psoriasis, Th1 and Th17-derived cytokines (such as TNFα and IL17A) may induce PARP2 in keratinocytes, which may turn down aromatase activity and estrogen synthesis in keratinocytes that may trigger NF-κB activation. However, the mechanistic details of such regulatory cascade remain to be determined.

A limitation of our study is the utilization of whole-body knock-out mice instead of knock-out mice with conditional deletion of PARP2 in keratinocytes or immune cells. Indeed, we cannot exclude the contribution of distinct skin cells or distant organs to the observed skin phenotype of PARP2^−/−^ mice; however, the mechanism we demonstrate in keratinocytes might be an important piece in the pathomechanism of psoriasis.

Currently, the most successful therapies against psoriasis are biologic agents targeting IL17A or TNFα, but there are several safety concerns about the usage of such therapies, and there is a constant search for better-tolerated solutions. Several PARP inhibitors are in clinical use for systemic application in tumor therapy, involving FDA-approved veliparib (Abbvie), rucaparib (Pfizer/Clovis), olaparib (KuDOS/AstraZeneca), niraparib (Merck/Tesaro), talazoparib (Lead/Biomarin/Medivation/Pfizer), and fluzoparib and pamibarib approved by the Chinese NMPA. These are pan-PARP inhibitors that equally target PARP1, PARP2, and even PARP3. Hence, selective targeting of PARP2 cannot be done by these inhibitors, and their effects may be affected by the concurrent inhibition of PARP1 and PARP3. However, here we show that talazoparib could recapitulate the effect of depletion or specific inhibition of PARP2 with UPF1069 on estradiol production. Given that the protective effect of genetic deletion or depletion of PARP2 in psoriasiform inflammation may probably be boiled down to increased estradiol synthesis of keratinocytes, our data might raise the potential of repurposing PARP inhibitors in the treatment of psoriasis. Still, the applicability of PARP inhibitors will have to be assessed in subsequent studies.

In summary, we highlighted a yet unknown mechanism by which PARP2 may be involved in inflammatory regulation and identified a potential targetable player in psoriasis. Our study may promote the development of PARP2 specific inhibitors and encourage that more studies be conducted on the elucidation of the role of PARP2 in inflammation and immune regulation.

## Supplementary Information

Below is the link to the electronic supplementary material.

Supplementary file1 (DOCX 22 KB)

## Data Availability

The authors made all primary data available at https://figshare.com/s/461e83a137b9b0080a14. The RNA-seq data is available in the BioProject database at https://dataview.ncbi.nlm.nih.gov/object/PRJNA889321?reviewer=bqb7td7m836rbh8hfgvqioh42e
