# Supplementary material for: PARP2 promotes inflammation in psoriasis by modulating estradiol biosynthesis in keratinocytes
Source: J Mol Med (Berl). 2023 Jun 23;101(8):987–99. doi: 10.1007/s00109-023-02338-z (PMC10400701; doi:10.1007/s00109-023-02338-z)
Supplement: Supplementary file 1 — Supplementary file1 (DOCX 22 KB) [file 109_2023_2338_MOESM1_ESM.docx]

**SUPPLEMENTARY MATERIAL**

**Materials and Methods**

All chemicals were from Merck KGaA (Darmstadt, Germany), unless stated otherwise. Recombinant human interleukin-17A (IL17A) was acquired from R&D Systems/Bio-Techne (7955-IL, Minneapolis, MN, USA). 5-bromo-2'-deoxyuridine (BrdU) was purchased from Abcam (ab142567; Cambridge, UK). Talazoparib was from Selleck Chemicals Gmbh (S7048, Planegg, Germany).

**Chromogenic in situ hybridization (CISH)**

Biopsies taken from scalp psoriasis lesions and from normal scalp skin of healthy individuals were used. CISH was performed on 5 µm thick sections of formalin-fixed paraffin embedded (FFPE) specimens with 100 nM double-digoxigenin labeled PARP2 specific (Custom LNA Detection Probe, Cat. No: 3395005-3DIG, LCD0165598-BKG, Qiagen, Hilden, Germany) or mRNA scramble negative control (Scramble-ISH Custom LNA Detection Probe lncRNA and mRNA, Cat. No: 3395085-3DIG LCD0000002-BDG, Qiagen) locked nucleic acid detection probes according to the manufacturer’s instructions. Sections were treated with 2x proteinase K solution (FFPE ISH Buffer Set, Cat. No:339450, Qiagen) for 15 min, then 100 nM of previously linearized (90ºC for 4 min) probe mix diluted in 1xISH buffer (miRNA ISH Buffer Set, Qiagen) was applied. Hybridization was performed at 53ºC with PARP2 specific and 57ºC with scramble negative control LNA probe for 60 min in a hybridization chamber (StatSpin ThermoBrite, Abbott Molecular, Chicago, IL, USA). For visualization alkaline phosphatase conjugated anti-DIG antibody (Roche, Basel, Switzerland) and 4-nitro-blue tetrazolium/5-bromo-4-chloro-indolylphosphate (NBT/BCIP, Roche) AP chromogen substrate was applied for 2 h at 30ºC. Slides were counterstained with liquid-stable nuclear fast red (VWR, Radnor, PA, USA), covered with Eukitt mounting medium (Sigma-Aldrich), coverglassed, and imaged using a Leica DM200LED (Leica, Wetzlar, Germany) microscope.

**Mice**

Homozygous *PARP2^-/-^* and littermate *PARP2^+/+^* mice on a C57BL/6J background from heterozygous crossings were used. Mice were subsequently bred at the animal facility of the University of Debrecen. No more than six mice were housed in each cage (standard block shape 365 × 207 × 140 mm, surface 530 cm2; 1284 L Eurostandard Type II. L from Techniplast) with Lignocel Select Fine (J. Rettenmaier und Söhne) as bedding. Mice had ad libitum access to water (sterilized tap water) and food (chow diet - Safe, Augy, France), and were kept under a 12/12 hr dark/light cycle. Only male mice were selected for the study at the age of 8 weeks, then mice were housed separately. Mice had paper tubes to enrich their environment. Cages were changed once a week, on the same day. The animal facility was overseen by a veterinarian. For the IMQ-model, hair was shaved off the back of littermate *PARP2^+/+^* and *PARP2^−/−^* male mice in a 2 × 2 cm area, subsequently, mice were treated daily with 62.5 mg of Aldara cream (5% imiquimod, Meda AB) or vehicle for four days to elicit psoriasiform lesions. For aromatase inhibition, 500 μM exemestane solution (in DMSO) was applied on the shaved skin of mice 30 minutes prior to Aldara treatment. The severity of the lesions, including induration, erythema and scaling, was scored daily by two experienced, blinded dermatologists on a scale from 0 to 4, where 0 denotes no symptoms and 4 denotes the most severe symptoms. On day 5, 2 hours prior to sacrifice, mice received 5-bromo-2-deoxyuridine (BrDU, 100 mg/ kg i.p.), then mice were sacrificed, and skin biopsies were taken for histology examination.

**Histology**

Immunohistochemistry analyses were performed on 4 μm thick sections cut from formalin fixed, paraffin embedded mouse or human skin samples, similar, as in Szántó et al., 2019. Briefly, heat-induced antigen retrieval was performed on formalin fixed samples. EDTA buffer (Reanal, Hungary, 1 mM, pH 8.0) was applied in microwave oven for 15 min to unmask epitopes of target proteins. Endogenous peroxidase activity was blocked with 3% H_2_O_2_ (Merck KGaA, Darmstadt, Germany) for 10 minutes. After blocking, tissue sections were incubated with specific primary antibodies (*RelA/NFkB p65* [p Ser276]: NB100-82086 and *CYP19A1:* NBP2-80415, Novus Biologicals; *Cytokeratin 10*: ab76318 and IL6: ab6672, Abcam; Involucrin: PRB140C, Covance). Protein expressions were detected with Mouse and Rabbit Specific HRP/DAB (ABC) Detection IHC kit (ab64264; Abcam) according to the manufacturer’s protocol. Negative controls were obtained by omitting the primary antibodies. For detection of 5-bromo-2-deoxyuridine, a BrdU immunohistochemistry kit was used (ab125306, Abcam).

**Cell culture**

Cellular studies were performed on HPV-Ker keratinocytes described in Tax et al., 2016 (doi: 10.2340/00015555-2154). The HPV-Ker cells were maintained in Keratinocyte serum-free medium (SFM) supplemented with Bovine Pituitary Extract (BPE) (50 μg/ml), human recombinant Epidermal Growth Factor (rEGF) (5 ng/ml) (Gibco, Thermo-Fisher Scientific, 17005042), 1% penicillin/streptomycin (Merck, P4333) and 1% L-glutamine solutions (Merck, 59202C) in 5% CO_2_ humidified air at 37 °C. For the differentiation of HPV-Ker keratinocytes, cells were seeded in 6-well plates (6×10^5^ cells/well) or petri dishes (TPP, 22,1cm², 1,4×10^6^ cells/dish). After three days, or in petri dish after four days, one day after the culture reached confluency, HPV-Kers were differentiated in culture media supplemented with 1.7 mM Ca^2+^ for three days.

**shRNA-mediated gene silencing**

PARP2 knocked-down HPV-Ker keratinocytes (shPARP2) were generated by stable transfection with a predesigned shRNA targeting PARP2 (target sequence: ACTATCTGATTCAGCTATTAG; clone ID: TRCN0000235599; ref. sequence: NM_005484) that was cloned into a pLKO.1 vector and packed in lentivirus carrier (Merck KGaA, Darmstadt, Germany). Negative control HPV-Kers (sc) were created by transfection with a non-target shRNA-containing pLKO.1 vector carrying transduction particles (SHC016H; Merck KGaA, Darmstadt, Germany). After transfection, puromycin-resistant cells were selected with 2.5 μg/ml puromycin and subsequently were maintained in Keratinocyte-SFM medium supplemented with human rEGF (5 ng/ml), BPE (50 μg/ml) (Gibco, Thermo-Fisher Scientific; 17005042), 1% L-glutamine (Merck, 59202C) and 2.5 µg/ml Puromycin Dihydrochloride (Gibco, Thermo Fisher Scientific, A1113803) at 37 °C in 5% CO_2_ atmosphere.

**RNA isolation and RT-qPCR**

HPV-Ker keratinocytes were seeded in 6-well plates (TPP, 2.5×10^5^ cells/well). Total RNA from cells was routinely isolated using TRIzol reagent (TR 118; Molecular Reserch Center, Inc., Cincinnati, OH, USA) according to the manufacturer’s instructions. The RNA samples used for RNA-sequencing were isolated with the RNeasy Kit from Qiagen (Hilden, Germany). RNA concentration and purity was assessed using a NanoDrop ND-1000 spectrophotometer (Thermo Fisher Scientific; Waltham, MA, USA). RNA samples were treated with Ambion DNase I (AM2222; Thermo Fisher Scientific), afterwards, 1 μg of DNAse-treated RNA samples were reverse transcribed using a High-Capacity cDNA Reverse Transcription Kit (4368813; Applied Biosystems, Foster City, CA, USA,). The RT-qPCR reactions were performed by using TaqMan assays and the TaqMan Gene Expression Master Mix protocol (Thermo Fisher Scientific, 4369016) in a Light-Cycler 480 Detection System (Roche Applied Science, Basel, Switzerland). As internal control, GAPDH (assay ID Hs02786624_g1) was used. The following TaqMan assays were used in the study: KRT10; assay ID Hs00166289_m1; IL23A; assay ID Hs00372324_m1; PARP2; assay ID Hs00193931_m1; IL6; assay ID Hs00174131_m1.

**RNA-sequencing (RNA-seq)**

To obtain global transcriptome data high throughput mRNA sequencing analysis was performed on Illumina sequencing platform. Total RNA sample quality was checked on Agilent BioAnalyzer using Eukaryotic Total RNA Nano Kit according to manufacturer’s protocol. Samples with RNA integrity number (RIN) value >7 were accepted for library preparation process. RNA-Seq libraries were prepared from total RNA using Ultra II RNA Sample Prep kit (New England BioLabs) according to the manufacturer’s protocol. Briefly, poly-A RNAs were captured by oligo-dT conjugated magnetic beads then the mRNAs were eluted and fragmented at 94-Celsius degree. First strand cDNA was generated by random priming reverse transcription and after second strand synthesis step double stranded cDNA was generated. After repairing ends, A-tailing and adapter ligation steps adapter ligated fragments were amplified in enrichment PCR and finally sequencing libraries were generated. Sequencing run were executed on Illumina NextSeq 500 instrument using single-end 75 cycles sequencing. For data analysis, raw sequencing data (fastq) was aligned to human reference genome version GRCh38 using HISAT2 algorithm and BAM files were generated. Downstream analysis was performed using StrandNGS software ([www.strand-ngs.com](http://www.strand-ngs.com)). BAM files were imported into the software DESeq algorithm was used for normalization. Moderated t-test with Benjamini-Hochberg FDR was used to determine differentially expressed genes between conditions. CytoScape v3.4 software with ClueGo v2.3.5. application was used for identifying over-represented Gene ontology (GO) terms. Two-sided hypergeometric test with Bonferroni step down correction was performed using the list of differentially expressed genes and GO Biological process database.

**Protein extraction and Western blotting**

HPV-Ker cells were seeded in petri dishes (TPP, 22,1cm², 2×10^6^ cells/dish). Cells were washed with phosphate buffered saline (PBS) solution and were then harvested in RIPA buffer (50 mM Tris, 150 mM NaCl, 1% Triton X-100, 0.5% sodium deoxycholate, 0.1% SDS, 1 mM EDTA, 1 mM Na_3_VO_4_, 1 mM NaF, 1 mM PMSF, protease inhibitor cocktail) on ice. DNA-protein complexes in cell lysates were disrupted by sonication (Branson Sonifier) for 3×20 pulses, 15-s intervals. Protein isolates were separated from debris by centrifugation for 10 minutes at 10,000 x g, 4°C.

For separation of cytosolic and nuclear protein fractions, cells were washed with ice-cold PBS, collected and centrifuged at 4 °C at 3,000 × g for 10 min. Pellets were homogenized in 600 μl buffer A (10 mM HEPES [pH 7.9]; 10 mM KCl, 1 mM EDTA, 1 mM EGTA, 1 mM DTT, protease inhibitors, and 0.5 % Nonidet-P) and were disrupted by aspirating through a 26-G needle 15 times using a 1 ml syringe. Lysates were vortexed and trypan blue staining was used to examine intact nuclei. Afterwards, the lysates were centrifuged at 4 °C at 15,000 × g for 1 min and the supernatant was used as cytosolic fraction. The pellets were washed in 400 μl buffer A and once again were needle-disrupted. After centrifugation at 4 °C at 15,000 × g for 1 min the pellets were resuspended in 400 μl buffer B (20 mM HEPES [pH 7.9]; 420 mM NACl, 0,5 mM EDTA, 0,5 mM EGTA, 1 mM DTT, and protease inhibitors) and were sonicated (Branson Sonifier) on ice (3X20 pulses, 15-s intervals), subsequently were centrifuged at 4 °C at 12,000 × g for 10 min, yielding the nuclear fractions. Protein concentration was determined using Pierce BCA protein assay kit (23225; Thermo Fisher Scientific; Waltham, MA, USA).

Protein lysates were boiled in 5x SDS sample buffer (310 mM Tris-HCl, pH 6.8, 50% glycerol, 10% SDS, 100 mM DTT, 0.01% bromophenol blue) supplemented with 2-mercaptoethanol for 10 minutes. Protein extracts (25-35µg) were separated by SDS-PAGE and transferred onto nitrocellulose membranes. Membranes were blocked with 5% BSA in TBS_Tween_ or 5% non-fat milk in TBS_Tween_ for 1 h at room temperature and incubated with primary antibodies overnight at 4 °C. Membranes were probed with a peroxidase-conjugated secondary antibody for 1 h at room temperature. Signals were visualized by enhanced chemiluminescence reaction and captured by ChemiDoc^TM^ Touch Imaging System (Bio-Rad, Hercules, CA, USA). Bands were quantified by densitometry using Image lab software 5.2.1 (Bio-Rad). The following antibodies were used in immunoblots: anti-PARP2 (Enzo Life Sciences, ALX-210-899-R100, 1:1000), anti-RelA/NFkB p65 (Novus Biologicals, NB100-82086, 1:1000), anti-CYP19A1 (Novus Biologicals, NBP2-80415; 1:1000), Recombinant anti-Cytokeratin 10 (Abcam, ab76318, 1:1000), anti-IL-23A (Abcam, ab45420, 1:1000), anti-β-actin-Peroxidase (Merck, A3854, 1:20000), anti-Histone H1 (Merck, 05-457, 1:1000), anti-mouse IgG, HRP-linked (Merck, A9044, 1:2000), anti-rabbit IgG, HRP-linked (Cell Signaling Technology, 7074S 1:1000).

**Cell cycle analysis of keratinocytes**

Cells were rinsed with PBS then trypsinized, washed with PBS, centrifuged at 2000 rpm for 5 min and fixed with 5 ml ice-cold 70% ethanol drop by drop while slowly vortexing. Afterwards, cells were incubated overnight at 4 °C, and centrifuged at 2000 rpm for 5 min. Cells were washed with staining buffer, then 100 µg/ml propidium iodide stock solution (Invitrogen Corporation, Carlsbad, CA, US., V13242B) was added to the staining buffer (1 µl/sample) and incubated for 20 min in dark. Cell cycle analysis was carried out by NovoCyte Flow Cytometer (ACEA Biosciences, Inc, Agilent, Santa Clara, CA, US).

**Mouse cytokine array**

Mouse skin samples were flash frozen in liquid nitrogen after excise. Before protein extraction, tissues were weighed and rinsed with PBS. Tissues were homogenized in PBS containing protease inhibitor cocktail (S8830, Merck KGaA, Darmstadt, Germany) using 5 mm stainless steel beads at 30 hertz for 8 min in a TissueLyser II device (Qiagen, Hilden, Germany). Afterwards, Triton X-100 was added to the samples to reach 1% final concentration. The samples were then centrifuged at 10,000 × g for 5 min at 4°C to remove any debris. The supernatant was saved, and protein content was quantified using a Pierce BCA protein assay kit (23225, Thermo Fisher Scientific; Waltham, MA, USA).

300 μg protein of each samples within a treatment group was pooled and used for the mouse cytokine array according to the protocol provided by the manufacterer (Proteome Profiler Array, No. ARY006, R&D Systems/Bio-Techne, Minneapolis, MN, USA). The immunoblot images were captured and visualized using the ChemiDoc™ Touch Imaging System (Bio-Rad Laboratories, Hercules, CA, USA) and the intensity of each spot in the images was analyzed using the CellProfiler for non-cell images analysis software (Broad Institute of MIT and Harvard, Cambridge, MA, USA).

**Determination of cytokine and estradiol concentration**

Supernatants were collected from HPV-Kers exposed to different treatments, and were analysed for human IL6, IL23A, IL17C and estradiol using commercially available ELISA kits (IL6 and estradiol: D6050 and KGE014, R&D Systems/Bio-Techne; Minneapolis, MN, USA; IL23A and IL17C: EH3270 and EH14723, Wuhan Fine Biotech Co., Ltd., Wuhan, China) according to the manufacturers’ protocols. IL23A and IL17C were not detected in the supernatants (data not shown).

Mouse skin protein samples were analysed for IL17A, TNFα and IL17C with commercially available ELISA kits according to the manufacturers’ instructions (IL17A and TNFα: EM1624 and EM0183, Wuhan Fine Biotech Co., Ltd., Wuhan, China; IL17C: ELK6078, ELK Biotechnology Co., Ltd., Wuhan, China).

**Immunocytochemistry**

Cells were grown on glass coverslips (4 × 10^4^ cells/coverslips) and exposed to various treatments. Next, cells were washed with PBS and fixed in ice-cold methanol for 10 min at -20°C. After fixation, the cells were washed PBS three times and permeabilized with 1% Triton X-100 in PBS for 10 min. Cells were blocked with 1% bovine serum albumin (BSA) in PBS at RT for 1 h. After blocking, cells were incubated with a primary antibody in 1% BSA dissolved in PBS (anti-IκBα L35A5 (Amino-terminal Antigen), Cell Signaling Technology, #4814, 1:400 dilution) overnight at 4 °C, then cells were washed three times with PBS and secondary antibody (Goat anti-Mouse IgG (H+L) Cross-Adsorbed Secondary Antibody, Alexa Fluor 488 Invitrogen, #A-11001, 1:500 dilution) was added to the cells for 1 hour at RT. Cell nuclei were visualized with DAPI (NucBlue Fixed Cell ReadyProbes Reagent, 1:10, R37606, Invitrogen Corporation, Carlsbad, CA, United States) for 10 min. Coverslips were adhered in Mowiol/Dabco solution (49:1). Confocal images were acquired with a Leica TCS SP8 confocal microscope and pictures were taken with LAS AFv3.1.3 software (Wetzlar, Germany).

**NF-κB activity assay**

Cells were seeded in 6-well plates (TPP, 1,5X10^5^ cells/well) and were exposed to various treatments. Proteins was extracted from the cells using Complete Lysis Buffer AM2 (provided from TransAM™ NFκB p65 Chemi Kits, 40097) and the protein content of supernatants was assessed using Bradford protein assay (Bio-Rad reagent). NF-κB p65 subunit activation was determined in 4 µg of whole-cell protein extracts from each sample using the TransAM™ NFκB p65 Chemi Kits (Active Motif, Carlsbad, CA, USA, 40097) according to the manufacturer’s instructions. The Jurkat treated with PMA and calcium ionophore nuclear extract provided with the kit was used as positive control for NFκB p65 activation. Luminescence was measured using a Spark 10M microplate reader (Tecan, Mannedorf, Switzerland).
